# (*Z*)-1-[(3-Cyano­phen­yl)iminiometh­yl]-2-naphtholate

**DOI:** 10.1107/S1600536809020224

**Published:** 2009-06-06

**Authors:** Yong-Feng Zhao, Jin-Ping Xiong, Yu Zuo

**Affiliations:** a College of Materials Science and Engineering, Beijing University of Chemical Technology, Beijing 100029, People’s Republic of China

## Abstract

The title compound, C_18_H_12_N_2_O, crystallizes in a zwitterionic form. The dihedral angle between the planes of the benzene ring and naphthalene ring system is 13.95 (5)°. An intra­molecular N—H⋯O inter­action results in the formation of a planar six-membered ring, which is oriented at dihedral angles of 13.50 (4) and 4.49 (4)° with respect to the benzene ring and naphthalene ring system, respectively. In the crystal structure, inter­molecular C—H⋯O and C—H⋯N inter­actions link the mol­ecules into a two-dimensional network. π–π contacts between the naphthalene systems [centroid–centroid distance = 3.974 (1) Å] may further stabilize the structure.

## Related literature

For the pharmacological activity of Schiff base compounds, see: Dao *et al.* (2000 ▶); Sriram *et al.* (2006 ▶). For the role played by Schiff base compounds in coordination chemistry related to magnetism, see: Chen *et al.* (2008 ▶); Weber *et al.* (2007 ▶). For related structures, see: Elmali *et al.* (2001 ▶); Yüce *et al.* (2006 ▶). For bond-length data, see: Allen *et al.* (1987 ▶).
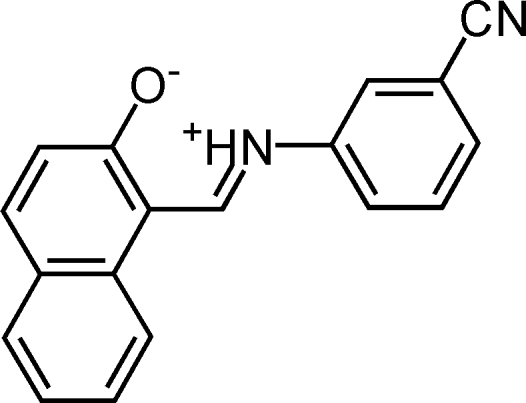


## Experimental

### 

#### Crystal data

C_18_H_12_N_2_O
*M* *_r_* = 272.30
Triclinic, 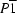

*a* = 7.8943 (16) Å
*b* = 9.1356 (18) Å
*c* = 9.4933 (19) Å
α = 83.97 (3)°
β = 84.41 (3)°
γ = 82.50 (3)°
*V* = 672.6 (2) Å^3^
*Z* = 2
Mo *K*α radiation
μ = 0.09 mm^−1^
*T* = 294 K
0.20 × 0.20 × 0.20 mm

#### Data collection

Rigaku SCXmini diffractometer
Absorption correction: multi-scan (*CrystalClear*; Rigaku, 2005 ▶) *T*_min_ = 0.976, *T*_max_ = 0.983
6177 measured reflections
2628 independent reflections
1146 reflections with *I* > 2σ(*I*)
*R*_int_ = 0.061

#### Refinement

*R*[*F* ^2^ > 2σ(*F* ^2^)] = 0.067
*wR*(*F* ^2^) = 0.195
*S* = 0.94
2628 reflections
190 parameters
H-atom parameters constrained
Δρ_max_ = 0.23 e Å^−3^
Δρ_min_ = −0.24 e Å^−3^

### 

Data collection: *CrystalClear* (Rigaku, 2005 ▶); cell refinement: *CrystalClear*; data reduction: *CrystalClear*; program(s) used to solve structure: *SHELXS97* (Sheldrick, 2008 ▶); program(s) used to refine structure: *SHELXL97* (Sheldrick, 2008 ▶); molecular graphics: *ORTEP-3 for Windows* (Farrugia, 1997 ▶) and *PLATON* (Spek, 2009 ▶); software used to prepare material for publication: *SHELXL97* and *PLATON*.

## Supplementary Material

Crystal structure: contains datablocks I, global. DOI: 10.1107/S1600536809020224/hk2700sup1.cif

Structure factors: contains datablocks I. DOI: 10.1107/S1600536809020224/hk2700Isup2.hkl

Additional supplementary materials:  crystallographic information; 3D view; checkCIF report

## Figures and Tables

**Table 1:** Hydrogen-bond geometry (Å, °)

*D*—H⋯*A*	*D*—H	H⋯*A*	*D*⋯*A*	*D*—H⋯*A*
N1—H1*A*⋯O1	0.86	1.87	2.562 (3)	136
C2—H2*A*⋯N2^i^	0.93	2.61	3.463 (3)	152
C6—H6*A*⋯O1^ii^	0.93	2.57	3.376 (3)	145
C17—H17*A*⋯N2^i^	0.93	2.62	3.522 (3)	163

Symmetry codes: (i) 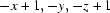; (ii) 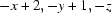.

**Fig. 1.**
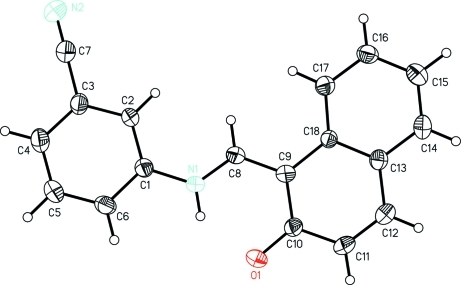
The molecular structure of the title molecule, with the atom-numbering scheme. Hydrogen bond is shown as dashed line.

**Fig. 2.**
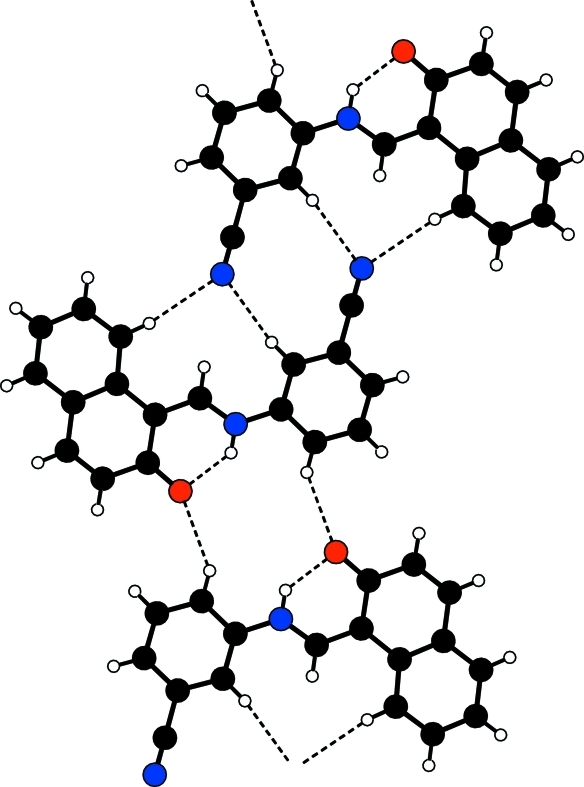
A partial packing diagram of the title compound. Hydrogen bonds are shown as dashed lines.

## supplementary crystallographic information

### Comment

Schiff base compounds have received considerable attention for many years, primarily due to various pharmacological activities, such as anticancer (Dao *et al.*, 2000) and anti-HIV (Sriram *et al.*, 2006) activities. In addition, Schiff base compounds play important roles in coordination chemistry related to magnetism (Weber *et al.*, 2007) and catalysis (Chen *et al.*, 2008). Generally, Schiff base compounds exhibit the phenol-imine and keto-amine forms. Another form of the Schiff base compounds is their zwitterionic form, and this form have been reported in the literature (Elmali, *et al.*, 2001). We report herein the crystal structure of the title compound.

The molecule of the title compound (Fig 1) is in a zwitterionic form. The bond lengths (Allen *et al.*,  1987) and angles are within normal ranges, and C8=N1 [1.304 (4) Å] and C10-O1 [1.287 (4) Å] bonds may be compared with the corresponding values [1.2954 (19) and 1.2946 (17) Å] in a similar zwitterionic structure (Yüce *et al.*, 2006). Phenyl and naphthalyl rings, A (C1-C6) and B (C9-C18), are, of course, planar and the dihedral angle between them is 13.95 (5)°. Intramolecular N-H···O interaction (Table 1) results in the formation of a planar six-membered ring C (O1/N1/C8-C10/H1A), which is oriented with respect to rings A and B at dihedral angles of 13.50 (4) and 4.49 (4) °, respectively.

In the crystal structure, intramolecular N-H···O and intermolecular C-H···O and C-H···N interactions (Table 1) link the molecules into a two-dimensional network (Fig. 2), in which they may be effective in the stabilization of the structure. The π–π contact between the naphthalyl rings, Cg2—Cg2^i^ [symmetry code: (i) -x, 1 - y, 1 - z, where Cg2 is centroid of the ring (C9-C13/C18)] may further stabilize the structure, with centroid-centroid distance of 3.974 (1) Å.

### Experimental

For the preparation of the title compound, 3-aminobenzonitrile (0.59 g, 5 mmol) and 2-hydroxynaphthalene-1-carbaldehyde (0.861 g, 5 mmol) were dissolved in ethanol (25 ml). The resulting mixture was heated to reflux for 6 h, and then cooled to room temperature. The solid product was collected by filtration. Crystals suitable for X-ray analysis were obtained on slow evaporation at room temperature.

### Refinement

H atoms were positioned geometrically, with N-H = 0.86 Å (for NH) and C-H = 0.93 Å for aromatic H and constrained to ride on their parent atoms, with U_iso_(H) = 1.2U_eq_(C,N).

### Crystal data

**Table:**

C_18_H_12_N_2_O	*Z* = 2
*M**_r_* = 272.30	*F*(000) = 284
Triclinic, *P*1	*D*_x_ = 1.345 Mg m^−^^3^
Hall symbol: -P 1	Mo *K*α radiation, λ = 0.71073 Å
*a* = 7.8943 (16) Å	Cell parameters from 4320 reflections
*b* = 9.1356 (18) Å	θ = 3.2–27.5°
*c* = 9.4933 (19) Å	µ = 0.09 mm^−^^1^
α = 83.97 (3)°	*T* = 294 K
β = 84.41 (3)°	Prism, yellow
γ = 82.50 (3)°	0.20 × 0.20 × 0.20 mm
*V* = 672.6 (2) Å^3^	

### Data collection

**Table:**

Rigaku SCXmini diffractometer	2628 independent reflections
Radiation source: fine-focus sealed tube	1146 reflections with *I* > 2σ(*I*)
graphite	*R*_int_ = 0.061
Detector resolution: 13.6612 pixels mm^-1^	θ_max_ = 26.0°, θ_min_ = 3.2°
ω scans	*h* = −9→9
Absorption correction: multi-scan (*CrystalClear*; Rigaku, 2005)	*k* = −11→11
*T*_min_ = 0.976, *T*_max_ = 0.983	*l* = −11→11
6177 measured reflections	

### Refinement

**Table:**

Refinement on *F*^2^	Primary atom site location: structure-invariant direct methods
Least-squares matrix: full	Secondary atom site location: difference Fourier map
*R*[*F*^2^ > 2σ(*F*^2^)] = 0.067	Hydrogen site location: inferred from neighbouring sites
*wR*(*F*^2^) = 0.195	H-atom parameters constrained
*S* = 0.94	*w* = 1/[σ^2^(*F*_o_^2^) + (0.0919*P*)^2^] where *P* = (*F*_o_^2^ + 2*F*_c_^2^)/3
2628 reflections	(Δ/σ)_max_ = 0.016
190 parameters	Δρ_max_ = 0.23 e Å^−^^3^
0 restraints	Δρ_min_ = −0.23 e Å^−^^3^

### Special details

**Table:**

Geometry. All e.s.d.'s (except the e.s.d. in the dihedral angle between two l.s. planes) are estimated using the full covariance matrix. The cell e.s.d.'s are taken into account individually in the estimation of e.s.d.'s in distances, angles and torsion angles; correlations between e.s.d.'s in cell parameters are only used when they are defined by crystal symmetry. An approximate (isotropic) treatment of cell e.s.d.'s is used for estimating e.s.d.'s involving l.s. planes.
Refinement. Refinement of *F*^2^ against ALL reflections. The weighted *R*-factor *wR* and goodness of fit *S* are based on *F*^2^, conventional *R*-factors *R* are based on *F*, with *F* set to zero for negative *F*^2^. The threshold expression of *F*^2^ > σ(*F*^2^) is used only for calculating *R*-factors(gt) *etc*. and is not relevant to the choice of reflections for refinement. *R*-factors based on *F*^2^ are statistically about twice as large as those based on *F*, and *R*- factors based on ALL data will be even larger.

### Fractional atomic coordinates and isotropic or equivalent isotropic displacement parameters (Å^2^)

**Table:**

	*x*	*y*	*z*	*U*_iso_*/*U*_eq_	
O1	0.9002 (3)	0.6505 (3)	0.2062 (2)	0.0608 (8)	
N1	0.7833 (3)	0.3998 (3)	0.2569 (3)	0.0439 (7)	
H1A	0.8270	0.4652	0.1974	0.053*	
N2	0.4237 (5)	−0.1364 (4)	0.3557 (4)	0.0852 (12)	
C1	0.7515 (4)	0.2689 (4)	0.2031 (3)	0.0421 (8)	
C2	0.6494 (4)	0.1697 (3)	0.2774 (3)	0.0452 (9)	
H2A	0.5984	0.1876	0.3675	0.054*	
C3	0.6234 (4)	0.0444 (4)	0.2177 (4)	0.0482 (9)	
C4	0.6973 (5)	0.0157 (4)	0.0832 (4)	0.0609 (11)	
H4A	0.6799	−0.0695	0.0437	0.073*	
C5	0.7971 (5)	0.1160 (4)	0.0093 (4)	0.0654 (11)	
H5A	0.8467	0.0985	−0.0812	0.079*	
C6	0.8240 (4)	0.2410 (4)	0.0675 (3)	0.0514 (10)	
H6A	0.8914	0.3077	0.0160	0.062*	
C7	0.5115 (5)	−0.0569 (4)	0.2954 (4)	0.0611 (11)	
C8	0.7525 (4)	0.4310 (3)	0.3886 (3)	0.0408 (8)	
H8A	0.7016	0.3630	0.4536	0.049*	
C9	0.7916 (4)	0.5615 (3)	0.4381 (3)	0.0405 (8)	
C10	0.8725 (4)	0.6662 (3)	0.3400 (4)	0.0433 (9)	
C11	0.9241 (5)	0.7903 (4)	0.3945 (4)	0.0576 (11)	
H11A	0.9789	0.8582	0.3323	0.069*	
C12	0.8969 (5)	0.8139 (4)	0.5334 (4)	0.0577 (10)	
H12A	0.9334	0.8968	0.5645	0.069*	
C13	0.8122 (4)	0.7125 (3)	0.6335 (4)	0.0424 (9)	
C14	0.7876 (4)	0.7374 (4)	0.7790 (4)	0.0533 (10)	
H14A	0.8261	0.8199	0.8091	0.064*	
C15	0.7077 (4)	0.6415 (4)	0.8761 (4)	0.0554 (10)	
H15A	0.6908	0.6590	0.9715	0.067*	
C16	0.6524 (4)	0.5185 (4)	0.8304 (4)	0.0509 (9)	
H16A	0.5981	0.4529	0.8957	0.061*	
C17	0.6766 (4)	0.4920 (4)	0.6902 (3)	0.0483 (9)	
H17A	0.6374	0.4087	0.6623	0.058*	
C18	0.7585 (4)	0.5863 (3)	0.5875 (3)	0.0367 (8)	

### Atomic displacement parameters (Å^2^)

**Table:**

	*U*^11^	*U*^22^	*U*^33^	*U*^12^	*U*^13^	*U*^23^
O1	0.083 (2)	0.0657 (17)	0.0347 (15)	−0.0255 (14)	0.0047 (13)	0.0010 (12)
N1	0.0520 (19)	0.0430 (17)	0.0370 (17)	−0.0146 (13)	0.0051 (13)	−0.0025 (14)
N2	0.120 (3)	0.064 (2)	0.076 (3)	−0.043 (2)	0.011 (2)	−0.010 (2)
C1	0.043 (2)	0.044 (2)	0.040 (2)	−0.0072 (16)	−0.0024 (16)	−0.0059 (17)
C2	0.057 (2)	0.044 (2)	0.035 (2)	−0.0102 (18)	0.0011 (16)	−0.0075 (16)
C3	0.057 (2)	0.042 (2)	0.048 (2)	−0.0068 (18)	−0.0062 (18)	−0.0107 (17)
C4	0.079 (3)	0.055 (2)	0.051 (3)	−0.008 (2)	−0.004 (2)	−0.018 (2)
C5	0.083 (3)	0.069 (3)	0.047 (2)	−0.018 (2)	0.010 (2)	−0.020 (2)
C6	0.053 (2)	0.061 (2)	0.040 (2)	−0.0129 (19)	0.0046 (17)	−0.0033 (18)
C7	0.080 (3)	0.051 (2)	0.054 (3)	−0.014 (2)	−0.001 (2)	−0.011 (2)
C8	0.040 (2)	0.044 (2)	0.038 (2)	−0.0066 (16)	0.0015 (15)	−0.0029 (16)
C9	0.039 (2)	0.041 (2)	0.041 (2)	−0.0065 (16)	−0.0007 (15)	0.0003 (16)
C10	0.052 (2)	0.0366 (19)	0.042 (2)	−0.0093 (16)	−0.0054 (17)	−0.0010 (16)
C11	0.070 (3)	0.047 (2)	0.058 (3)	−0.0253 (19)	−0.001 (2)	0.003 (2)
C12	0.068 (3)	0.050 (2)	0.060 (3)	−0.022 (2)	−0.003 (2)	−0.007 (2)
C13	0.041 (2)	0.042 (2)	0.046 (2)	−0.0029 (16)	−0.0066 (16)	−0.0074 (17)
C14	0.053 (2)	0.055 (2)	0.056 (3)	−0.0066 (19)	−0.0103 (19)	−0.020 (2)
C15	0.058 (2)	0.064 (3)	0.043 (2)	−0.003 (2)	0.0004 (18)	−0.009 (2)
C16	0.057 (2)	0.051 (2)	0.043 (2)	−0.0085 (18)	0.0046 (17)	−0.0013 (18)
C17	0.052 (2)	0.045 (2)	0.048 (2)	−0.0097 (18)	0.0029 (17)	−0.0074 (18)
C18	0.0366 (19)	0.0372 (19)	0.0377 (19)	−0.0076 (15)	−0.0012 (14)	−0.0074 (15)

### Geometric parameters (Å, °)

**Table:**

O1—C10	1.287 (4)	C9—C10	1.431 (4)
N1—C1	1.409 (4)	C9—C18	1.453 (4)
N1—C8	1.304 (4)	C11—C10	1.416 (4)
N1—H1A	0.8600	C11—C12	1.350 (5)
N2—C7	1.142 (4)	C11—H11A	0.9300
C2—C1	1.384 (4)	C12—H12A	0.9300
C2—C3	1.376 (4)	C13—C12	1.435 (4)
C2—H2A	0.9300	C13—C14	1.414 (4)
C4—C3	1.387 (5)	C13—C18	1.403 (4)
C4—C5	1.377 (5)	C14—H14A	0.9300
C4—H4A	0.9300	C15—C14	1.370 (4)
C5—H5A	0.9300	C15—C16	1.382 (5)
C6—C1	1.392 (4)	C15—H15A	0.9300
C6—C5	1.369 (5)	C16—H16A	0.9300
C6—H6A	0.9300	C17—C16	1.369 (4)
C7—C3	1.458 (5)	C17—C18	1.399 (4)
C8—H8A	0.9300	C17—H17A	0.9300
C9—C8	1.406 (4)		
			
C1—N1—H1A	117.0	O1—C10—C9	122.5 (3)
C8—N1—C1	126.0 (3)	O1—C10—C11	119.7 (3)
C8—N1—H1A	117.0	C11—C10—C9	117.8 (3)
C2—C1—N1	123.1 (3)	C10—C11—H11A	118.7
C2—C1—C6	119.0 (3)	C12—C11—C10	122.6 (3)
C6—C1—N1	117.9 (3)	C12—C11—H11A	118.7
C1—C2—H2A	120.1	C11—C12—C13	120.8 (3)
C3—C2—C1	119.8 (3)	C11—C12—H12A	119.6
C3—C2—H2A	120.1	C13—C12—H12A	119.6
C2—C3—C4	121.1 (3)	C14—C13—C12	120.0 (3)
C2—C3—C7	119.2 (3)	C18—C13—C12	119.9 (3)
C4—C3—C7	119.6 (3)	C18—C13—C14	120.0 (3)
C3—C4—H4A	120.7	C13—C14—H14A	119.6
C5—C4—C3	118.6 (4)	C15—C14—C13	120.8 (3)
C5—C4—H4A	120.7	C15—C14—H14A	119.6
C4—C5—H5A	119.6	C14—C15—C16	119.1 (3)
C6—C5—C4	120.9 (4)	C14—C15—H15A	120.5
C6—C5—H5A	119.6	C16—C15—H15A	120.5
C1—C6—H6A	119.7	C15—C16—H16A	119.6
C5—C6—C1	120.5 (3)	C17—C16—C15	120.8 (3)
C5—C6—H6A	119.7	C17—C16—H16A	119.6
N2—C7—C3	179.7 (4)	C16—C17—C18	122.1 (3)
N1—C8—C9	123.9 (3)	C16—C17—H17A	119.0
N1—C8—H8A	118.0	C18—C17—H17A	119.0
C9—C8—H8A	118.0	C13—C18—C9	118.5 (3)
C8—C9—C10	118.8 (3)	C17—C18—C13	117.1 (3)
C8—C9—C18	120.7 (3)	C17—C18—C9	124.4 (3)
C10—C9—C18	120.4 (3)		

### Hydrogen-bond geometry (Å, °)

**Table:**

*D*—H···*A*	*D*—H	H···*A*	*D*···*A*	*D*—H···*A*
N1—H1A···O1	0.86	1.87	2.562 (3)	136
C2—H2A···N2^i^	0.93	2.61	3.463 (3)	152
C6—H6A···O1^ii^	0.93	2.57	3.376 (3)	145
C17—H17A···N2^i^	0.93	2.62	3.522 (3)	163

Symmetry codes: (i) −*x*+1, −*y*, −*z*+1; (ii) −*x*+2, −*y*+1, −*z*.
